# Some Interesting Cases and Notes

**Published:** 1909-12-04

**Authors:** John Cleland

**Affiliations:** Emeritus Professor of Anatomy in the University of Glasgow


### SOME INTERESTING CASES AND NOTES.

### REMINISCENCES OF PRACTICE.

By JOHN CLELAND, M.D., LL.D., F.R.S.; Emeritus Professor of Anatomy in the University of Glasgow.

Being asked for some recollections of practice in my earlier days, I venture to put together a few reminiscences which stand out prominently in my mind, and which I have occasionally alluded to in my anatomical teaching. I may begin with a curious case of

Partial Fracture of the Sternum.

I was once asked by a medical colleague in Galway to see along with him a man who had fallen backwards from a cart loaded with hay, and who was showing grave disturbance of respiration and complaining of acute pain. The patient was breathing with great difficulty, and the pain of which he complained was located in one spot on the left edge of the sternum, near the lower end, and unbearably sore when touched. The left side of the chest had a distended appearance and was tympanitic all over, and there was no fever. These circumstances gave the key to the nature of the lesion. In trying to keep his balance on the top of the hay the patient would fill his lungs and hold his breath to give fixity to the recti abdominis muscles and to the sterno-mastoids. Under the force of the spasmodic contraction of these muscles the sternum had given way on the left side; the inflated extremity of the inferior lobe of the lung, being pressed against the opening gap in the bone, had got caught between the fractured edges as they came together in the expiration after the effort and had been torn, so that the air escaped from the wounded lung into the pleural cavity, producing pneumothorax. I succeeded in repressing the desire of my friend to administer a course of calomel and opium, and I had the satisfaction of seeing the patient quite recovered within a very few days without any treatment of importance.

Fracture of the sternum from muscular action would appear to be a lesion of considerable rarity. Hyrtl (*Topogr. Anat.*, I., 431) writes thus: "That the sternum can be broken across by great throwing backwards of the trunk seems to me not possible, since it is not the thoracic but the lumbar part of the column which can be bent backwards. Nevertheless, the occurrence of transverse fracture through falling on the back from a considerable height is indisputable. I have seen with Scarpa fracture of the sternum united by flat callus. Simultaneous vehement contraction of the muscles attached to the upper and lower end of the sternum (sterno-mastoid and rectus abdominis) have in this way occasioned transverse fracture, which must be considered as rupture not from compression by a squeezing force, but by excessive pulling asunder." This distinction drawn by Hyrtl may at first sight appear to be an over-refinement, but it is thoroughly accurate.

Excision of the Elbow and Knee Joints.

Some reminiscences occur to me with reference to these operations which I may be allowed to record in cursory fashion. In the days when I attended the clinic of Mr. Syme, his excisions of the elbow joint and his remarks on the operation were most instructive. In his hands that operation became a marked success, the wound healing up quickly, and the patient being provided with a moveable joint and a useful hand. But when afterwards, in winter 1855-6, I saw this excision performed by Paul Broca, afterwards celebrated both as an anthropologist and in connection with the pathology of aphasia, both I and friends who were with me were constrained to note how far behind the Parisians then were with regard to that valuable operation. We were surprised at the great length of time which was expected to elapse before the cure could be established. But our surprise was removed when we saw the method of operation pursued; two of its most marked features being that the ulnar nerve was carefully dissected out and held aside with a hook, and that the head of the radius was removed by means of a chain-saw passed round the neck. I may mention that the chain-saw was an invention of Dr. Jeffray, Professor of Anatomy in the University of Glasgow, before Dr. Allen Thomson whom I afterwards succeeded. It was an ingenious invention, but I should think that it is never now used. Jeffray published and figured it in 1806 in a book in which he reproduced some of the earliest papers on excision of joints, namely, several by Mr. Park, of Liverpool, and also a translation of Moreau's memoir on the same subject, in which full justice is done to Park. But excision of the elbow joint was first brought to perfection by Syme, who pointed out not only an easy and rapid method of operating, but the importance of preserving mobility. The appreciation of this requisite in excision of the elbow joint made Syme, however, sceptical as to the utility of excision of the knee, in which immobility and solid strength are desired, and he discountenanced the action of his colleague in the Edinburgh Infirmary, Richard Mackenzie, when that surgeon performed the operation, and was believed to be the first to do so in this country. I remember the case and the long lapse of time before the patient could make any use of his limb. Yet Park had excised the knee in 1781, and he mentions that the operation took place on July 2, and that the patient began to walk on crutches on December 1. A delay occurred afterwards from a fall owing to one of the crutches giving way, but he subsequently laid aside crutches, got a strong and useful leg, and, being a sailor, was able to go aloft and perform all the duties of a seaman. It is needless to say that a much more rapid recovery may now be expected; but it may not be amiss to mention some points which, being attended to, aid a much speedier recovery than, perhaps, is yet as common as it ought to be. The most important thing is not to injure the posterior ligament. The late Mr. Butcher, of Dublin, invented a saw to enable the surgeon to remove the head of the tibia from behind forwards; but not only is this unnecessary, it is a plan which necessitates injury to the posterior ligament. In the cases which I had I ceased pressing the saw further back as soon as it had begun to score the shell of compact bone behind, and with a little force I elevated the piece to be removed and completed its separation by using the scalpel. By this means the periosteum and the attachment of the posterior ligament were left intact, and a strong fibrous floor was left separating the wound from the loose tissues in the popliteal space. Drainage was further facilitated by carrying the outer side of the flap back as far as the posterior edge of the external lateral ligament. I used no long splint, but fitted behind the knee a folded piece of perforated zinc, little more than a foot in length and well padded; while in front I placed another folded piece of perforated zinc so bent that in the middle part of its length it stood up over the face of the wound like the handle of a pot lid. The padded upper and lower ends of the splint so formed were then bandaged so that the femur and tibia were grasped between this fore splint and the splint behind, while the face of the wound was left free in its whole extent for dressing. In the first case which I had I was rather surprised and alarmed, on going to see the patient an hour or more afterwards, to find him lying on the side on which the operation had been performed; but my mind was immediately set at rest by finding that he was perfectly comfortable and the limb quite straight, the outer end of the flap pendant, giving free drainage to the whole interior of the wound. This was in the Workhouse Hospital in Galway, in which, in those days, the bed consisted of a sack filled with straw, and the only special antiseptic precaution consisted in having poured into the wound oil containing one part of carbolic acid to sixty of oil. It was some years afterwards that, driving in the country on an Irish jaunting car, I heard a loud halloaing, and found a young fellow in charge of sheep who was madly running after me with a stiff leg; and this proved to be my grateful patient, who could not let me pass without thanking me.

### Stricture of the Urethra.

Writing about Mr. Syme's clinic in old days, it is natural that my mind should revert to his charming mastery of the catheter. It was a treat to see him, with one hand in his pocket, introduce the instrument and pass it with the utmost ease, then similarly pass others until he had passed with smooth sweep into the bladder the largest that the stricture would allow. This to a student contrasted with the manipulations of other surgeons, who used both hands and sometimes betrayed the tense condition of their muscular system by the expression of their faces; and when I came to have hospital patients of my own I felt anxious to discover the secret of his facility. Imitating him, I put my left hand in my pocket, stood relaxed and leisurely, and was surprised how easily the instrument would slide through even a considerable stricture, and felt rather inclined to regret that the great teacher had not explained that the placing of the left hand in contact with the perinæum was the cause of much of the difficulty which many practitioners experience, and that the slightest discomposure on the part of the operator interferes with the freedom of passage of the instrument. I remember his once being baffled in passing an instrument, and his adding to his dictum that no stricture was impermeable the important addition, "provided that the surgeon has sufficient dexterity," which in this instance he admitted that he himself had not.

Syme held that stricture was always in front of the bulb; and undoubtedly it is so in the great majority of cases; but I had once, and only once, a case in which beyond all possibility of doubt the stricture was confined to the membranous portion of the urethra. The opening in the triangular ligament could be distinctly felt, and beyond this the instrument was caught in the constricted membranous portion. It is at the triangular ligament that practitioners often find difficulty, even with a healthy urethra, in passing the instrument further; and it ought to be explained to students more frequently than it is that they ought to recognise the sensation of the point of the instrument resting against a shallow depression, and that especially in patients who have not been previously catheterised it is well to hold the catheter quietly in this position, when after a little time relaxation of the constrictor will be felt taking place, and the instrument seems as if it went onwards of its own accord. I would not mention such an everyday occurrence were it not that over and over again I have observed good men deficient in acquaintance with it. But when real stricture of the membranous part occurs the sensation is quite different, resembling the grasp of the ordinary prebulbar stricture, only more liable to bleed.

The great benefit to be got from copaiba in troublesome stricture was brought home to me by a patient who declared that he had at one time suffered from stricture, and getting no relief at the hands of his doctor applied to a quack, who gave him something which, from its taste, he knew to have copaiba in it, and felt immensely benefited. I acted on the suggestion when an opportunity occurred, and soon found that administration of a few five-drop doses of copaiba invariably resulted in the instrument passing more easily the next day.

### Stone in the Bladder.

It is true that I have only once operated for stone in the bladder, and that the lateral operation then in favour seems now to be given up on account of the results which antiseptic surgery gives to the supra-pubic operation. But my own case had peculiarities which may be of interest. An old man came into hospital who had been examined both in Belfast and by the late Dr. Fleming, of Dublin, without a stone being discovered. By a happy chance I struck a stone distinctly, and I operated, notwithstanding that I failed again to strike it when the patient was put on the operating table. Recalling an advice given by Syme, when I got my fingers into the bladder I divided the muscular ring at the neck of the bladder, and was surprised how much advantage it gave by opening up the interior. I took out two uric acid calculi about the size of small nutmegs, and could find no more. But, before the patient was removed, I examined them and found that one of them had on one side a close-grained, flat surface, while the rest of it was rounded and rough; while the other presented two such flat surfaces at an angle one to the other, suggesting that it had rubbed against two other stones. I therefore placed the patient again in position, but on the most careful search could find no more. The patient did well, but after a few days he began to pass by the urethra curious fragments of uric acid, presenting on one side a smooth convex surface, and on the other a concave surface covered with beautiful rhomboid crystals of uric acid standing out from it. These fragments must have been broken portions of a lining of a cystic depression behind the prostate. Probably one of the calculi removed had occupied this cyst and afterwards been dislodged, while the emptied hollow became lined with a new crust which had been broken up by muscular contractions during the healing of the wound. The patient left hospital in good health, but I heard that he reappeared in Belfast a year or more afterwards with the old complaint and died in hospital. The two calculi are preserved in the Anatomical Museum of the University of Glasgow, but unfortunately the fragments of the hollow deposit were lost.

I may mention a curious urethral case. A man came into hospital with a stricture which had led to extravasation of urine. Two sloughs had formed, one on each side on the groin, without there being any urinary infiltration in either perinæum or scrotum. The stricture was attended to, the inguinal sloughs healed, and the patient left hospital cured. This passage of the urine on to the front of the abdomen without damage to the perinæum and scrotum seems very remarkable, and can only be explained in one way, namely, that the urine failed to injure the accelerator urinæ and was carried forward so as to escape from beneath the anterior edge of the tendon into the groin above the attachment of Scarpa's fascia to Poupart's ligament.

